# Periodontal regeneration in swine after cell injection and cell sheet transplantation of human dental pulp stem cells following good manufacturing practice

**DOI:** 10.1186/s13287-016-0362-8

**Published:** 2016-09-09

**Authors:** Jingchao Hu, Yu Cao, Yilin Xie, Hua Wang, Zhipeng Fan, Jinsong Wang, Chunmei Zhang, Jinsong Wang, Chu-tse Wu, Songlin Wang

**Affiliations:** 1 Molecular Laboratory for Gene Therapy & Tooth Regeneration, Beijing Key Laboratory of Tooth Regeneration and Function Reconstruction, Capital Medical University School of Stomatology, Tian Tan Xi Li No. 4, Beijing, 100050 China; 2 Department of Experimental Hematology, Beijing Institute of Radiation Medicine, 27 Taiping Road, Beijing, 100850 People’s Republic of China; 3 Beijing SH Bio-tech Corporation, Beijing, 100070 China; 4 Department of Biochemistry and Molecular Biology, Capital Medical University School of Basic Medical Sciences, Beijing, 100069 China

**Keywords:** Dental pulp stem cells, Cell injection, Cell sheet, Periodontal bone regeneration

## Abstract

**Background:**

Periodontitis, one of the most prevalent infectious diseases in humans, results in the destruction of tooth-supporting tissues. The purpose of the present study is to evaluate the effect of cell injection and cell sheet transplantation on periodontal regeneration in a swine model.

**Methods:**

In the present study, human dental pulp stem cells (hDPSCs) were transplanted into a swine model for periodontal regeneration. Twelve miniature pigs were used to generate periodontitis with bone defects of 5 mm in width, 7 mm in length, and 3 mm in depth. hDPSCs were obtained for bone regeneration using cell injection or cell sheet transplantation. After 12 weeks, clinical, radiological, and histological assessments of regenerated periodontal tissues were performed to compare periodontal regeneration treated with xenogeneic cell injection and cell sheet implantation.

**Results:**

Our study showed that translating hDPSCs into this large animal model could significantly improve periodontal bone regeneration and soft tissue healing. After 12 weeks, both the hDPSC sheet treatment and hDPSC injection significantly improved periodontal tissue healing clinically in comparison with the control group. The volume of regenerative bone in the hDPSC sheet group (52.7 ± 4.1 mm^3^) was significantly larger than in the hDPSC injection group (32.4 ± 5.1 mm^3^) (*P* < 0.05). The percentage of bone in the periodontium in the hDPSC injection group was 12.8 ± 4.4 %, while it was 17.4 ± 5.3 % in the hDPSC sheet group (*P* < 0.05).

**Conclusion:**

Both hDPSC injection and cell sheet transplantation significantly regenerated periodontal bone in swine. The hDPSC sheet had more bone regeneration capacity compared with hDPSC injection.

**Electronic supplementary material:**

The online version of this article (doi:10.1186/s13287-016-0362-8) contains supplementary material, which is available to authorized users.

## Background

Periodontitis, one of the most prevalent infectious diseases in humans, results in the destruction of tooth-supporting tissues such as bone, periodontal ligaments, and cementum [1]. Several regenerative approaches, including guided tissue regeneration [2], application of biological mediators such as enamel matrix derivative (EMD) [3], and other scaffold-based techniques [4], were proposed to treat periodontal disease, and favorable results were obtained in clinical trials and animal models. Based on recent progress in tissue engineering, ex vivo expanded mesenchymal stem cells (MSCs) are used in regenerative medicine because of their potential to differentiate into multiple lineages [5–9]. Previously, we generated a swine model of periodontitis [10]. In this model, we induced significant periodontal tissue regeneration using periodontal ligament stem cells (PDLSCs) mixed with hydroxyapatite/tricalcium phosphate (HA/TCP) scaffolds [10], allogeneic PDLSC sheets [11], and vitamin C (Vc)-treated PDLSC sheets [12]. However, inflamed autogenous PDLSCs had markedly dysfunctional immunomodulatory properties [13]; moreover, sources of PDLSCs are limited, largely impeding the clinical application of this approach. Compared to other adult tissue sources, dental pulp stem cells (DPSCs) are an easily accessible type of adult dental stem cell. In addition, they are capable of differentiating into at least three distinct cell lineages: osteo/odontogenic, adipogenic, and neurogenic [14]. Thus, DPSCs are a new and appropriate cell source for periodontal tissue regeneration [15]. In the present study, we transplanted human DPSCs (hDPSCs) via cell injection and cell sheets, following good manufacturing practice (GMP) [16], for the treatment of periodontitis in miniature pigs to evaluate the periodontal tissue regeneration capacity of both approaches. Because of the close similarity between minipigs and humans in terms of histology and functions of the orofacial tissues, this experimental design may yield important preclinical information about the application of stem cell-based therapy for treating periodontitis in humans.

## Methods

### Experimental animals

Twelve inbred male Wuzhishan miniature pigs, 12 months old and weighing 30–40 kg, were obtained from the Institute of Animal Science of the Chinese Agriculture University (Beijing, China). The present study was approved by the animal care and use committee of Capital Medical University (Reference number: AEEI-2015-089). The animal care and experimental procedures were carried out in accordance with guidelines of the Beijing Experimental Animal Management Ordinance. All surgical procedures were performed under general anesthesia using a combination of 6 mg/kg ketamine chloride and 0.6 mg/kg xylazine (intramuscular injection) before the experimental procedures.

### Culture of hDPSCs under GMP

The hDPSCs were cultured in a GMP-compliant facility with ISO 8 clean room standards equipped with class II and class III bio-safety cabinets and all other standard tissue culture equipment. The xenobiotic-free cell culture reagents included: animal-free origin collagenase (Worthington Biochemical Corporation, Lakewood, NJ, USA), CELLstart, EZPassage Tool, HBSS-Ca/Mg free, D/F12, TrypLE, xeno-free B27, N2 supplement, MSCGM-CD and ProFreeze CDM (Invitrogen/Gibco, Carlsbad, CA, USA), human serum (Innovative Research, Inc., Novi, Michigan, USA), basic fibroblast growth factor-2 (bFGF-2; Peprotech, Rocky Hill, NJ, USA), TeSR2 which includes high levels of bFGF-2 together with transforming growth factor-β (TGF-β; Stem Cell Technologies, Vancouver, BC, Canada), and Nutristem Stemedia (Stemgent, San Diego, USA), which consists of human recombinant insulin, human serum albumin, transferrin, human fibroblast growth factor, and TGF-β.

Normal human impacted third molars were collected from adults (19–29 years of age) at the Dental Clinic of the Beijing Stomatological Hospital under approved guidelines set by the Research Ethical Committee of Capital Medical University, China. All patients gave their written informed consent to participate. Tooth surfaces were cleaned and cut around the cementum-enamel junction using sterilized dental fissure burs to reveal the pulp chamber. The pulp tissue was gently separated from the crown and root and then digested in collagenase for 1 h at 37 °C. Single-cell suspensions were obtained by passing the cells through a 70-μm strainer (Falcon; BD Labware, Franklin Lakes, NJ, USA). All cells used in this study were from passage 3–4, which were 15–20 divisions of the primary hDPSCs. The same passage of hDPSCs was used for each experiment. The characterization of MSCs, including the expression profiles of surface molecules, colony forming unit fibroblasts (CFU-F) assay, and multi-lineage differentiation, was performed as previously reported [10] (Additional file 1: Figure S1).

### Making hDPSC sheets

The hDPSCs (1.0 × 10^5^) were subcultured in 60-mm dishes. According to a previous report [12], 20 mg/ml Vc was added to the culture medium for the duration of the experiment. The cells became confluent after 2–3 days in culture. Confluent cells were cultured for 7–10 days until the cells at the edge of the dishes wrapped, which implied that cell sheets had formed and could be detached. Samples of the hDPSC sheet were processed for cell count, histological examination, transmission electron microscopy (TEM), and scanning electron microscopy (SEM).

### Generation of the periodontitis model and hDPSC administration

Twelve miniature pigs were used to generate periodontitis lesions of the first molars as previously reported [10, 11] for a total of 24 defects. After clinical assessment, a mucoperiosteal flap was raised and alveolar bone was removed using a surgical bur to create experimental periodontal bone defects in the mesial region of the maxilla and mandibular first molars. The alveolar bone defect was 5 mm in width, 7 mm in length, and 3 mm in depth, and notch-shaped marks were made on the root surface at the level of the top of the alveolar crest and the floor of the defect (Additional file 2: Figure S2A). Each defect was used as the region of interest for statistical analysis. Three walls of the bone defect were alveolar bone, and the root surface in the bone defect (Additional file 2: Figure S2A) was instrumented using Gracey curettes (Shanghai Kangqiao Dental Instruments Factory, Shanghai, China) to remove all periodontal ligaments as well as cementum to expose the dentin surface between two notch-shaped marks. These defects were then randomly assigned to three groups, each consisting of eight defects in four miniature swine. The shape of the bone defect was detected by periodontal probe (bone sounding). The tip of the needle was stopped at the bottom of the bone defect beneath the periosteum. hDPSCs were injected into the bottom of the periodontal bone defects beneath the periosteum (Additional file 2: Figure S2). The hDPSC injection group was injected with approximately 1 × 10^7^ hDPSCs in 0.6 ml of 0.9 % NaCl at three sites (approximately 0.2 ml per site): the mesial side of the bone defect, the distal side of the bone defect, and the middle of the bone defect. The control group was injected with 0.9 % NaCl at the same sites as the hDPSC injection group. In order to demonstrate the aggregation of hDPSCs at the bone defect, we also performed iodinated contrast media injection following the same procedure as hDPSC injection. In the hDPSC sheet group, hDPSC sheets were prepared for tissue regeneration in vivo based on our previous report [12]; briefly, 1.0 × 10^5^ hDPSCs were cultured in 60-mm dishes with 20.0 μg/ml Vc for 10–15 days to make a sheet, which contained approximately 1 × 10^7^ hDPSCs/sheet. The hDPSC sheet was washed with 0.9 % NaCl repeatedly to remove culture medium as well as residual Vc. The surgical procedure and placement of the hDPSCs sheet was performed as follows: Periodontal defects were surgically prepared on the mesial roots of the bilateral mandibular first molars. An intracrevicular incision was made on the buccal aspect, from distal of the forth premolar to the mesial of the second molar. Following elevation of the buccal mucoperiosteal flap, root planning was performed using Gracey curettes (Shanghai Kangqiao Dental Instruments Factory, Shanghai, China). A single layer of hDPSC sheet was then placed on the denuded root in the bone defect. The mucoperiosteal flap was repositioned and sutured tightly at the cemento-enamel junction (CEJ) covering the grafted cell sheet with Gore-Tex suture (Gore-Tex Suture® CV-5, W. L. Gore and Associates, Inc., Flagstaff, AZ, USA). At 12 weeks after transplantation, all animals were sacrificed, and samples were harvested and fixed with 4 % paraformaldehyde (Sigma-Aldrich Corp.) and assessed histologically.

### SEM observation

Ex vivo expanded hDPSC sheets grown for 7–10 days were fixed using 2.5 % glutaraldehyde in 0.1 mol/l sodium cacodylate buffer (pH 7.2) for 2 h at 4 °C. After washing with sodium dimethylarsenate buffer, the cells were post-fixed in 1 % osmium tetroxide, dehydrated with gradient alcohol, and then incubated with isoamyl acetate. After gold coating, five samples were examined using a Hitachi S-520 scanning electron microscope (Hitachi, Tokyo, Japan).

### TEM observation

Harvested hDPSCs and hDPSC sheets were fixed using 2.5 % glutaraldehyde in 0.1 mg/ml sodium cacodylate buffer (pH 7.2) for 2 h at 4 °C. After fixation, three samples were rinsed three times with 0.1 mol/l sodium cacodylate buffer (pH 7.2) for 0.5 h. The samples were post-fixed in 2 % osmium tetroxide, washed for 1 h, dehydrated in a graded ethanol series, and embedded in Epon 812 resin according to the manufacturer’s instructions. Serial 0.5-mm sections were cut and examined using a light microscope (BHS-RFK; Olympus, Japan) after staining with 2 % toluidine blue for 5 min. For TEM analysis, nine 70-nm sections were cut, stained with 2 % uranylacetate for 30 min and 2 % lead citrate for 5 min, and observed with a JEM1010 transmission electron microscope (JEOL, Tokyo, Japan).

### Detection and quantitative analysis of implanted hDPSCs in local periodontal tissue

At 12 weeks after transplantation, all animals were sacrificed and the samples from the experimental area were harvested and underwent DNA extraction and PCR analysis to quantify the numbers of hDPSCs in recipients. Genomic DNA for PCR analysis was prepared from local alveolar bone and soft tissues in bone defects using a QIAamp DNAmini kit (Qiagen, Valencia, CA, USA). The human β-globin gene and the endogenous miniature pig receptor-associated protein at the synapse (RAPSYN) gene were amplified with Premix Ex Taq (probe qPCR) (Takara Bio, Otsu, Japan). For human β-globin, the forward primer was 5’-GTGCACCTGACTCCTGAGGAGA-3’, the reverse primer was 5’-CCTTGATACCAACCTGCCCAGG-3’, and the probe, labeled with fluorescent reporter and quencher, was 5’-FAM-AAGGTGAACGTGGATGAAGTTGGTGG-TAMRA-3’. For miniature pig RAPSYN, the forward primer was 5’-CTCACTTGTTCTTTCTTCTG -3’, the reverse primer was 5’-AGCCAGTGTTAGTACCTA-3’, and the probe was 5’-FAM-TATCTGACCCACCCATCCTGC-TAMRA-3’.

### Clinical and radiological evaluations

At week 12, the probing depth (PD) and attachment loss (AL) were evaluated on all experimental teeth pre-transplantation (week 0) and post-transplantation. The PD values were established with a Williams periodontal probe (Shanghai Kangqiao Dental Instruments Factory, Shanghai, China). At 4 weeks after operation and 12 weeks after cell implantation, these defects were examined by computed tomography (CT; Siemens, Erlangen, Germany) to monitor the defect shape. The scanning conditions were: 120 kV, 250 mA, 0.75 mm slice thickness, and 3-s slice acquisition time (Additional file 2: Figure S2C). Data were stored using the Dicom 3.0 standard. Three-dimensional CT imaging was reconstructed to assess the tissue regeneration. Dicom format default images were introduced into Mimics software. Threshold values were set according to the Bone (CT) Scale in Mimics. Three-dimensional models were reconstructed using Optimal, a setting in Mimics. An ASCII stereolithography (STL) file of the bone was imported into Geomagic Studio.

### Quantitative and histological assessment of regenerated periodontal tissues

At 12 weeks after transplantation, all animals were sacrificed and the samples from the experimental area were harvested and fixed with 4 % formaldehyde. The heights of new bone regeneration were measured using a Williams periodontal probe: the distance from the top of the newly formed bone to notch-shaped CEJ marks made during the operation was scaled. Each sample was measured at three different positions from the buccal to the lingual side. Mean values were recorded, and the heights of new bone regeneration were 7 mm minus mean values. The proportion of bone volume occupying the virtual spaces of the defect was measured, allowing quantitative comparisons among the three groups. Then the harvested samples were assessed histologically. Five sites of the hDPSC injection group, five sites of the control group and five sites of the hDPSC sheet group were subsequently decalcified with buffered 10 % edetic acid (pH 8.0) for 8 to 12 weeks and embedded in paraffin. Sections were deparaffinized and stained with hematoxylin and eosin (H&E). For histopathological assessment, buccal-lingual-direction sections of the experimental region were cut. Sections (5 μm) were deparaffinized and stained with H&E. For quantification of bone formation, the extent of bone within each section was analyzed semiquantitatively by NIH Image J as described previously [17]; five representative areas at × 5 magnification in each group were used. The area of bone formation was expressed as the percentage of bone in the periodontium in the sections.

### Statistical analysis

All statistical calculations were performed with SPSS13 statistical software. The statistical unit was used as the region of interest. Quantitative data were expressed as the mean ± standard deviation (SD) and analyzed by one-way analysis of variance (ANOVA). Statistical significance was determined by the independent sample test or analysis of variance. Comparison between the groups was made by analyzing data with the post-hoc method. Statistical significance was set at a level of *P* < 0.05. Multiple comparisons between the three groups was performed using the Student-Newman-Keuls (S-N-K) test method.

## Results

### Characterization of hDPSCs and hDPSC sheets under GMP

**Fig. 1:**
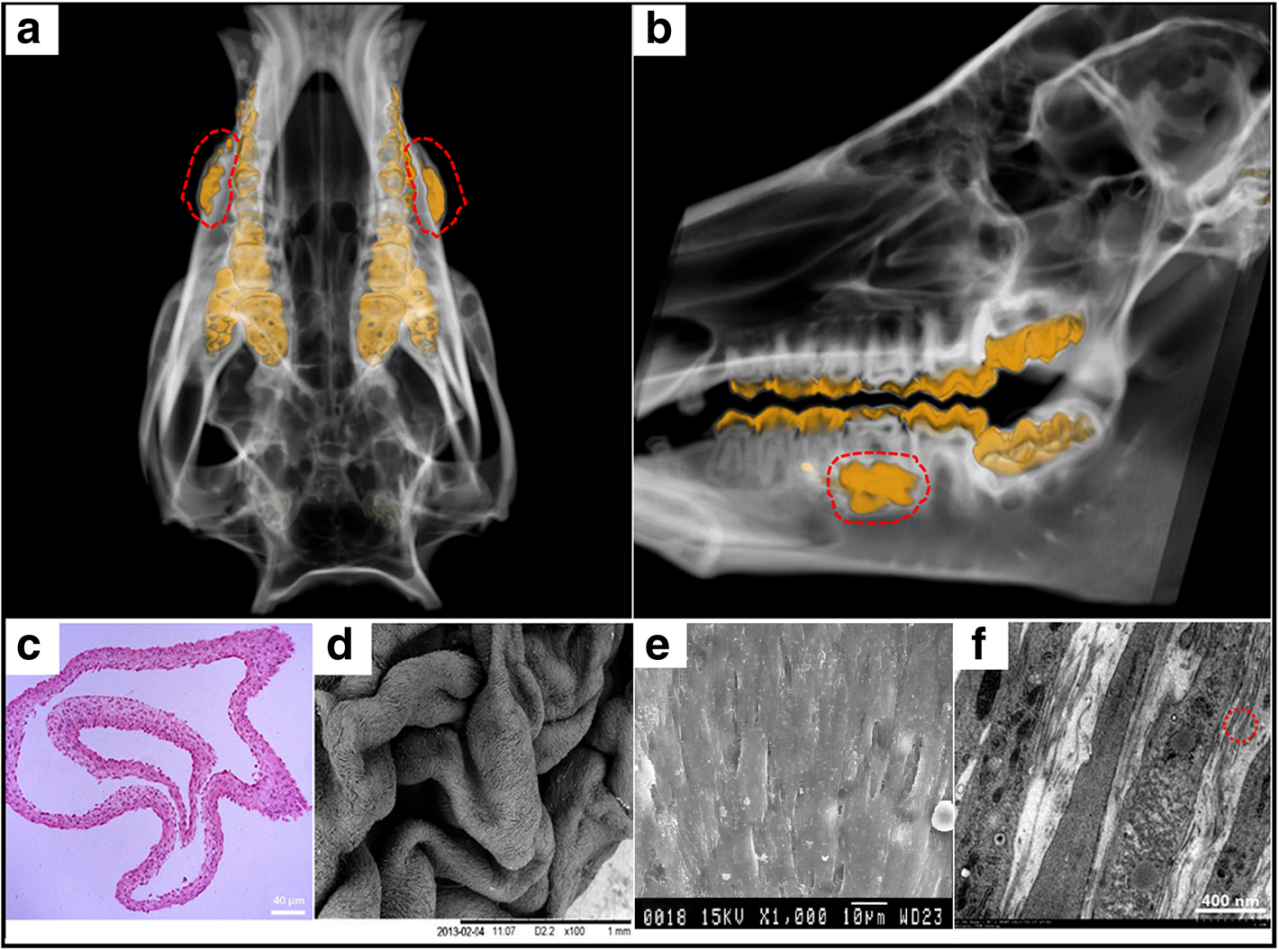
Location of injected hDPSCs and hDPSC sheet under good manufacturing practice. After the injection of iodinated contrast media, axial (**a**) and sagittal (**b**) CT slice images demonstrate the aggregation of contrast medium in mandible bone defects (*yellow blocks* in *red dotted line*), which indicated the location of injected hDPSCs beneath the periosteum. **c** The hDPSCs, induced with 20.0 μg/mL Vc, form a morphologically complete cell sheet; the cell sheet contained five or six layers and the cells contacted with each other tightly. **d** Scanning electron microscopy (SEM) of a hDPSC sheet. Morphologically, the hDPSC sheet was wave-shaped. **e** SEM of a hDPSC sheet (×1000), all cells contacted each other tightly. **f** Transmission electron microscopy (TEM) of a hDPSC sheet. Endogenous ECM (*ellipse*) was observed between cells

The cells isolated from dental pulp tissue within each colony were characterized by a typical fibroblast-like morphology under GMP conditions. Dental pulp tissue initially yielded a few cells appearing between 2 and 8 days. The colony forming efficiency was 9–12 % at 14 days of culture. At passage four (after 35 ± 5 days), each cultured sample (about 0.2 g of pulp tissue) yielded about 1 × 10^7^ cells. Flow cytometry analysis of hDPSCs revealed expression of the cell markers CD73, CD105, CD90, and CD146, but not HLA-DR and CD45 (Additional file 1: Figure S1D). A CT image showed that injection of iodinated contrast media was localized in the root surface and periodontal bone defects beneath the periosteum (Fig. 1a and b; Additional file 2: Figure S2). H&E staining revealed that the harvested whole hDPSC sheet contained five or six layers of cells, and was spread as a membrane-like structure with a uniform thickness (Fig. 1c). Morphologically, the hDPSC sheet was wave-shaped, all cells contacted each other tightly under SEM (Fig. 1d), and the secreted extracellular matrix (ECM) was around them. Exocytosis vesicles were observed near the secreted fiber base, demonstrating the sheet’s cell proliferation and differentiation characteristics (Fig. 1e). TEM of a hDPSC sheet showed exocytotic vesicles near the plasma membrane. These data indicated that the obtained hDPSC sheet preserved the intercellular junctions and endogenous ECM, and retained their cellular phenotypes.

### Both the hDPSC sheet and local injection of hDPSCs enhanced periodontal soft tissue healing and bone regeneration in swine

**Fig. 2:**
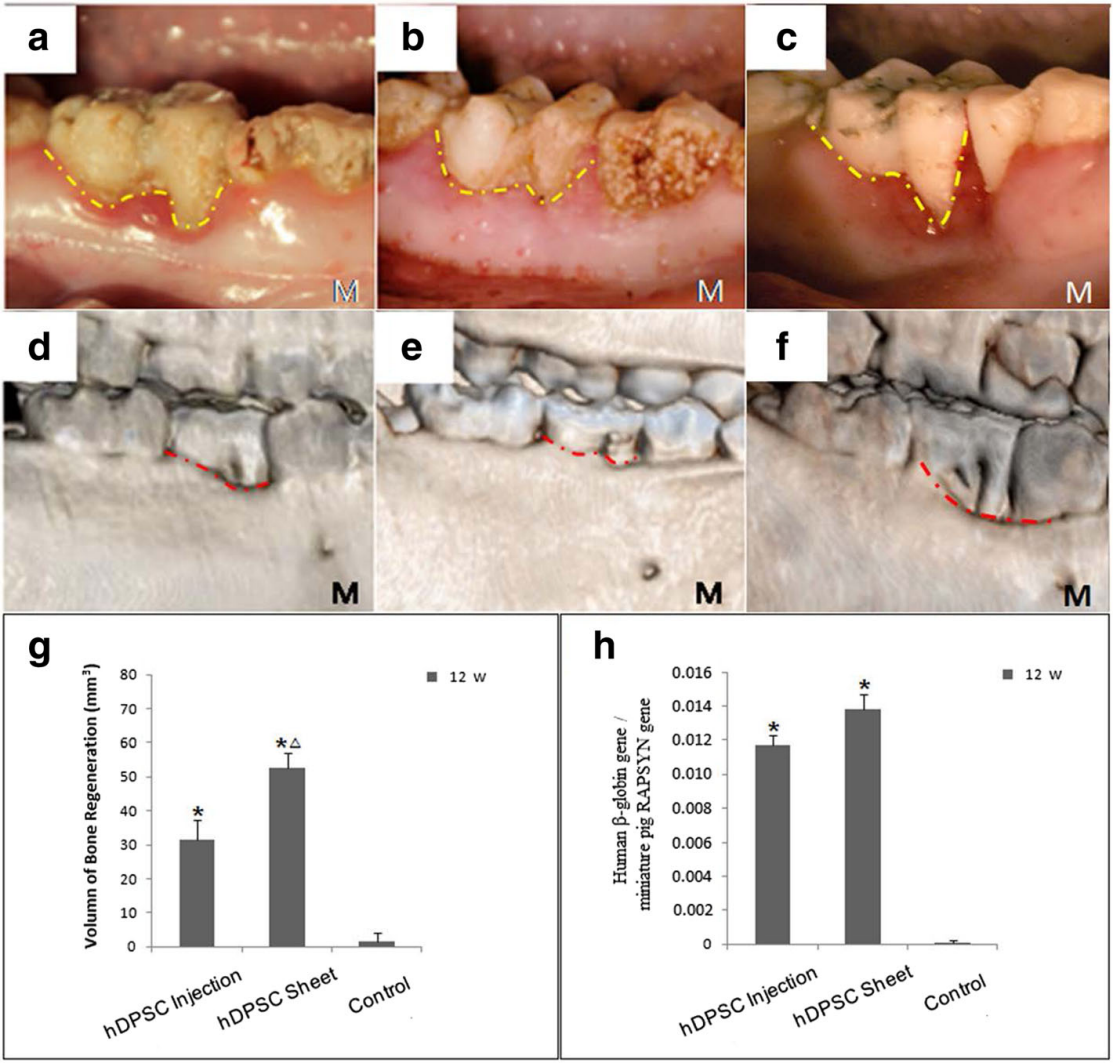
Healing of periodontal defects mediated by hDPSCs. **a**–**c** Intraoral photographs indicated that, 12 weeks after transplantation, marked periodontal soft tissue formation was found in the hDPSC injection group (the injection was performed without flap elevation), but could not restore soft tissues to healthy levels (**a**) (*yellow dotted line*). Periodontal soft tissue healing mediated by the hDPSC sheet (**b**) was close to the normal tissue level (*yellow dotted line*). Only limited periodontal soft tissues were recoverd in the control group (**c**) (*yellow dotted line*). **d**–**f** Three-dimensional CT images revealed marked bone formation in the hDPSC injection group (**d**), hDPSCs sheet group (**e**) after cell transplantation, and limited bone regeneration in the control group (**f**) (*red dotted lines*). **g** The bone regeneration volumes were larger in the hDPSC sheet group and hDPSC injection group compared with the control group (*P < 0.05). The bone regeneration volume was larger in the hDPSC sheet group than the hDPSC injection group (^△^ *P* < 0.05). **h** Genomic DNA was extracted from periodontal soft tissue and alveolar bone in the bone defect at 12 weeks after hDPSC implantation. Quantitative PCR was used to detect the human β-globin gene, and the results were normalized to the miniature pig receptor-associated protein at the synapse (*RAPSYN*) gene. Results are shown as means ± SD. *P < 0.05, versus control group. Statistical significance was evaluated by analysis of variance. All error bars represent SD (*n* = 5). *hDPSC* human dental pulp stem cell, *M* Mesial, *W* weeks

**Fig. 3:**
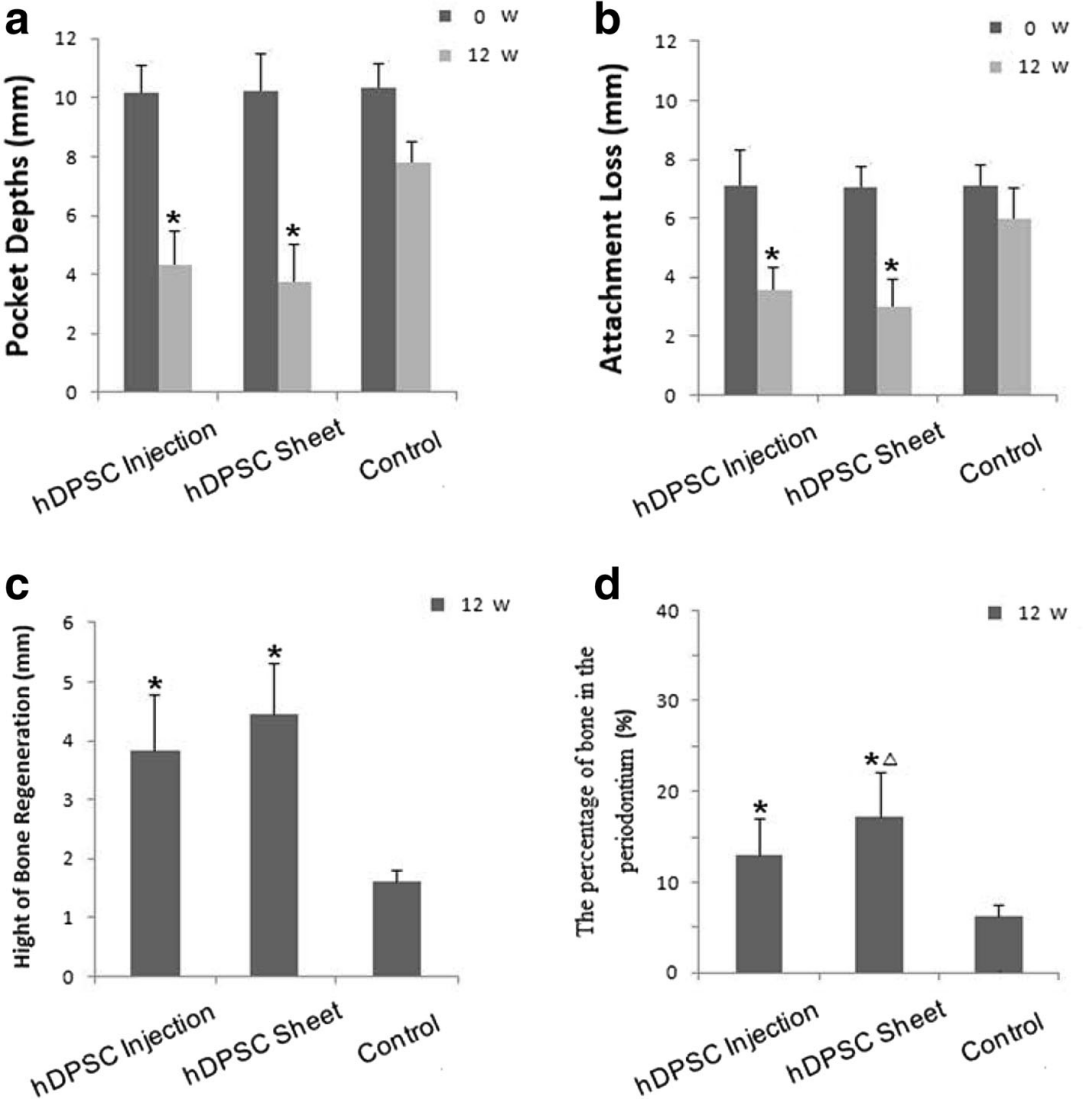
Clinical and bone qualitative assessments of regenerated periodontal tissues mediated by hDPSC transplantation in miniature pigs. **a**, **b** Clinical assessments of the periodontal situation in the three groups. At week 0, there was no significant difference in PD (**a**) and AL (**b**) among the three groups. However, at 12 weeks post-transplantation, the PD (**a**), and AL (**b**) values were significantly improved in the hDPSC injection and hDPSC sheet groups compared with the control group. Data are expressed as the mean ± SD (mm). The differences in clinical indexes at each time point among the three groups were analyzed using one-way analysis of variance. The pairwise comparisons were analyzed using the Bonferroni method (*P <0.05, *n* = 5). **c** The bone regeneration length was highest in the hDPSC sheet group, while the height of bone regeneration in the hDPSC injection group was also higher than the control group, indicating there was more bone tissue regeneration in the hDPSC injection and hDPSC sheet groups than in the control group (*P < 0.05). **d** Semi-quantitative analysis shows the amount of bone formation in each group. The percentage of bone in the periodontium was larger in the hDPSC sheet group and hDPSC injection group compared with the control group (*P < 0.05); the bone area was larger in the hDPSC sheet group than that in the hDPSC injection group (^△^ *P* < 0.05). Statistical significance was evaluated by analysis of variance. All error bars represent SD (*n* = 5). *hDPSC* human dental pulp stem cell, *W* weeks

**Fig. 4:**
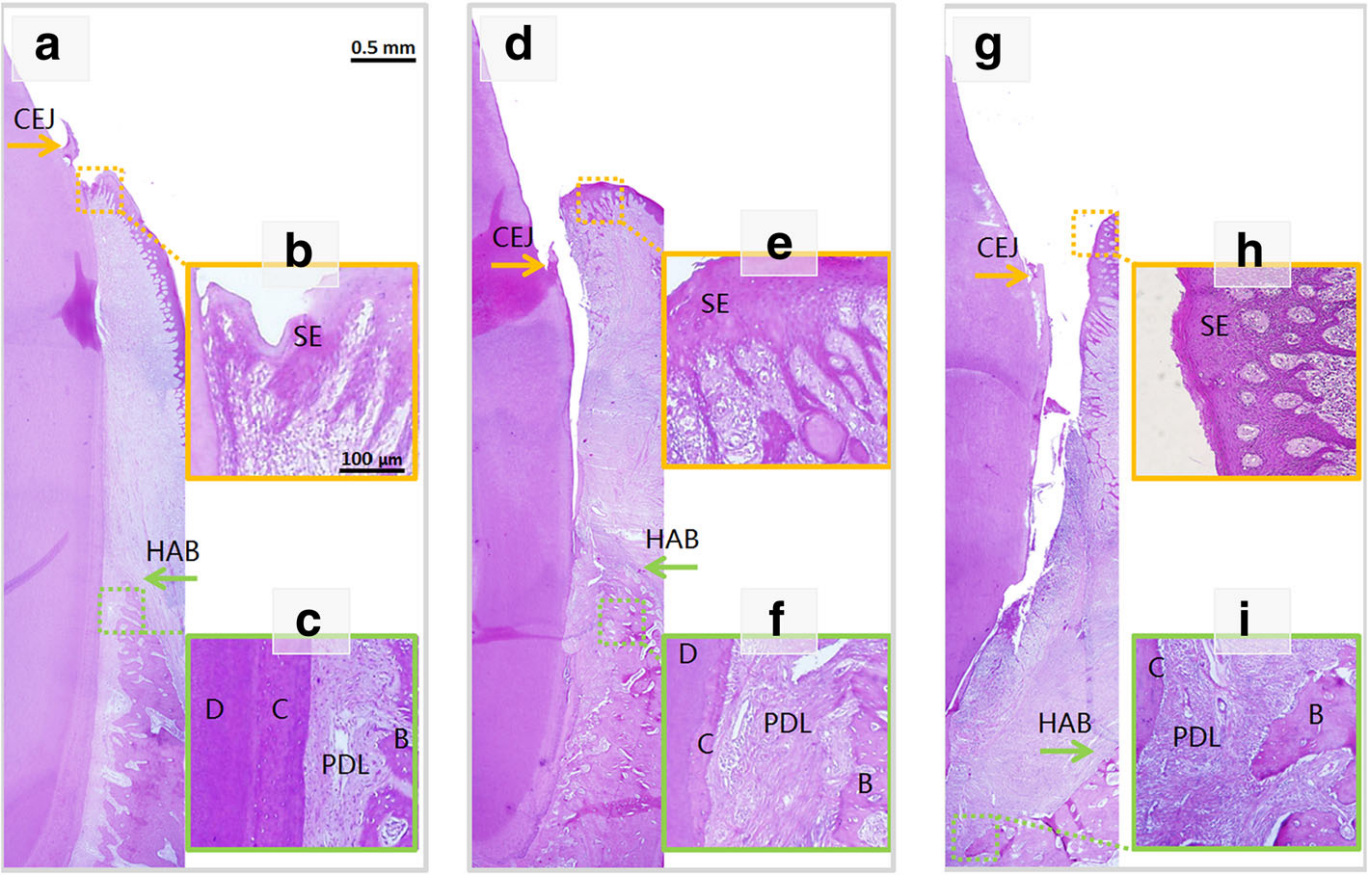
Histopathological assessment of periodontal bone regeneration by H&E staining. New periodontal bone regeneration in the periodontal defects of the hDPSC injection group (**a**) and hDPSC sheet group (**d**). New bone was regenerated in the periodontal defect area in the hDPSC injection group (**c**) and hDPSC sheet group (**f**). The alveolar bone heights in the hDPSC sheet group and hDPSC injection group were much larger than those in the control group (**i**). *B* bone, *C* cementum, *CEJ* cemento-enameljunction, *d* dentin, *HAB* height of alveolar bone, *hDPSC* human dental pulp stem cell, *PDL* periodontal ligament, *SE* sulcular epithelium. However, deep periodontal pockets and shortages of new bone remainedin the control group (**g**). Much thicker sulcular epithelia and enlarged epithelial pegs were evident in the control group (**h**) compared with *hDPSC* injection (**b**) and *hDPSC* sheet group (**e**)

We generated periodontitis lesions in miniature swine and then transplanted hDPSC sheets or disassociated cells for tissue regeneration. The animals were sacrificed at 12 weeks post-transplantation. Intraoral photographs showed that, 12 weeks after transplantation, marked periodontal tissue healing was found in the hDPSC injection group (Fig. 2a) and the hDPSC sheet group (Fig. 2b). There were only limited reattached periodontal tissues in the control group (Fig. 2c). Three-dimensional CT images indicated marked bone regeneration in the hDPSC injection (Fig. 2d) and hDPSC sheet (Fig. 2e) groups after cell transplantation, while limited bone formation was seen in the control group (Fig. 2f). Three-dimensional models at 12 weeks post-transplantation and pre-transplantation were reconstructed using Mimics (Additional file 3: Figure S3). The regenerated bone volume was calculated (Fig. 2g). At 12 weeks post-transplantation, the AL was 3.1 ± 0.6 mm in the hDPSC sheet group, 3.5 ± 0.6 mm in the hDPSC injection group, and 5.7 ± 0.5 mm in the untreated control group (Fig. 3b). Statistical analysis indicated that both hDPSC sheet treatment and hDPSC injection significantly improved periodontal soft tissue healing in comparison with the control group (Fig. 3a and b). The heights of new bone regeneration were significantly higher in the hDPSC sheet group and hDPSC injection group than in the control group (Fig. 3c). The CT scan and three-dimensional CT imaging showed that the volumes of regenerative alveolar bone in the hDPSC sheet group and hDPSC injection group were 52.7 ± 4.1 mm^3^ and 32.4 ± 5.1 mm^3^, respectively, which were significantly larger than the volume in the control group (1.8 ± 2.3 mm^3^, Fig. 2g). At 12 weeks after cell implantation, experimental tissues were also sectioned in the buccal-lingual direction and stained with H&E to provide a view of the entire section. Image J semi-quantitative analysis showed the percentage of bone in the periodontium in the hDPSC injection group and hDPSC sheet group were 12.8 ± 4.4 % and 17.4 ± 5.3 %, respectively, which was significantly larger than the volume in the control group (7.2 ± 2.0 %) (Fig. 3d). New bone was regenerated in the hDPSC sheet group (Figs. 3d and 4d) and hDPSC injection group (Figs. 3d and 4a). A new cementum-like layer from the height of alveolar bone (HAB) to almost the CEJ was observed in the hDPSC injection group (Fig. 4a) and hDPSC sheet group (Fig. 4d). This structure is missing in the control group (Fig. 4g). There was new attachment of Sharpy's fibers in the hDPSC sheet group (Fig. 4f) and hDPSC injection group (Fig. 4c), but attachment was irregular in the control group (Fig. 4i). Positive human β-globin expression was found in the tissues from the cell implantation group, while negative expression was found in the control group (Fig. 2h).

### Comparison of cell sheet transplantation and cell injection in periodontal regeneration

Although local hDPSC injection significantly improved periodontal tissue regeneration compared with the control group, intraoral photographs at 12 weeks post-injection showed it could not restore tissues to healthy levels (Fig. 2a). The height of periodontal alveolar bone in the hDPSC injection group was 3.8 ± 0.5 mm, while it was 4.5 ± 0.3 mm in the Vc-mediated hDPSC sheet group (Fig. 3c). The percentage of bone in the periodontium in the hDPSC sheet group was 17.4 ± 5.3 % while it was 12.8 ± 4.4 % in the hDPSC injection group (Fig. 3d). Thus, the periodontal bone regeneration capacity was greater for the hDPSC sheet.

## Discussion

In the present study, we investigated for the first time the feasibility of using hDPSC injection and hDPSC sheet implantation for the treatment of periodontitis in a large animal model. Regeneration of new bone was detected in both the hDPSC injection group and hDPSC sheet group 12 weeks after transplantation. In the control group, periodontal defects were largely restored by fibrous tissue and epithelia, and limited irregular new attachment was observed. These results suggested that the hDPSC injection and hDPSC sheet implantation contributed significantly more to periodontal tissue regeneration than in the control group. Intraoral photographs at 12 weeks post-injection showed hDPSC injection did not restore tissues to a healthy level compared with the hDPSC group. CT scan analyses also demonstrated that the volume of periodontal alveolar bone in the hDPSC sheet group was significantly larger than that in the hDPSC injection group. Therefore, the hDPSC sheet was more effective in periodontal tissue regeneration.

The ultimate goal for periodontal therapy is the simultaneous regeneration of alveolar bone, cementum, and periodontal ligament. However, what conventional periodontal therapies, including non-surgical treatments (such as scaling, root planning) and periodontal flap surgery, can achieve is no better than arresting the disease process—the tissue healing ends up in the formation of a long weak junctional epithelium instead of periodontal attachment. As a result, conventional periodontal therapies do not lead to periodontal regeneration, but rather to reattachment via establishment of long junctional epithelium [18]. As conventional periodontal regeneration methods remain insufficient to stimulate complete and functional periodontal regeneration, various regenerative therapies such as guide tissue regeneration (GTR) have been routinely utilized together in clinical practice [19, 20]. The GTR procedure is regarded as the first generation of periodontal regeneration strategy, which functions by placing a cell-occlusive membrane around the periodontal defect in order to avoid epithelial downgrowth and to promote the proliferation of undifferentiated progenitor cells in the remaining periodontal ligament tissues. The GTR procedure was improved by the development of new materials; for example, a novel membrane material [21]. The bilayered membrane induced greater periodontal regeneration than traditional membranes in a class II furcation defect in dogs. However, patients with chronic periodontitis are generally middle-aged and older people, and obviously their progenitor cells of periodontal tissue are much less active compared with young donors in terms of the differentiation capacity and cementum/periodontal ligament-like tissue formation [22]. Besides, this procedure often involves autograft, allograft, or xenograft transplantation, such as freeze-dried bone allograft. There are only a few areas of the body conducive for harvesting autograft tissue. The problem associated with these allo-/Xeno-biomaterial approaches is that the host’s immune system rejects what it identifies as foreign tissues [23–25]. Autograft, allograft, and xenograft transplantation may also require internal fixation which bears the risk of infection like any other invasive procedure. Therefore, the outcomes of GTR are limited and associated with poor clinical predictability [26]. Enamel matrix derivative (EMD) is regarded as another candidate protein mixture that induces mesenchymal cells to differentiate into new periodontal tissues [27, 28]. EMD has been demonstrated to promote periodontal regeneration to a certain degree, although its true effect remains to be determined [29–32]. Further well-controlled clinical trials are needed to justify the clinical application of EMD.

Recent studies have focused on the possible application of stem cells and tissue engineering to regenerate the periodontal structure. Together with the recent progress in tissue engineering, cell-based therapies have developed as a foundation for periodontal regenerative therapy [33]. MSCs are considered as a suitable cell source for the treatment of periodontitis not only for their capacity to regenerate different types of tissues, but also for their paracrine potential, secreting large quantities of growth factors and anti-inflammatory cytokines such as TGF-β and interleukin (IL)-10 which play important roles in systemic and local immunomodulation [34, 35]. Moreover, MSCs have little immunogenicity, enabling the use of allogeneic cells [34]. In our previous studies, we demonstrated that allogeneic transplantation of MSCs into swine periodontal defect models did not induce immunorejection [36]. Furthermore, we found that prostaglandin E2 (PGE2) played a crucial role in PDLSC-mediated immunomodulation and periodontal tissue regeneration both in vitro and in vivo. PDLSCs suppressed B-cell activation through cell-to-cell contact, which was mostly mediated by programmed cell death protein 1 and programmed cell death 1 ligand 1 [36]. In the present study, human β-globin gene was still detectable at 12 weeks after hDPSC application. However, it is not sufficient to confirm the presence of live hDPSCs with the reported PCR result only. Tissue regeneration may be affected by the species of MSC origin, immunological status of the host, and presence or absence of inflammation [37, 38]; the detailed mechanism of how hDPSCs mediate periodontal regeneration in the pig model still needs to be further investigated.

Our previous studies have indicated that transplanted PDLSCs [10–12], stem cells from exfoliated deciduous teeth (SHED) [39] and bone marrow-derived MSCs (BMMSCs) [40] can regenerate periodontal tissues, including periodontal ligament and alveolar bone. Among the optional MSCs, DPSCs have a richer tissue source, and higher proliferating and colony-forming properties than BMMSCs [41], and are easier to isolate than PDLSCs. In addition, the DPSC harvesting procedure from the extracted third molars is non-invasive. In the present study, we used xenogeneic DPSCs cultured under GMP guidelines for periodontal tissue regeneration and investigated the feasibility of using hDPSC injection and hDPSC sheet implantation for the treatment of periodontitis. Cell injection therapy has been the most common approach for treating a variety of diseases [42, 43]. In our previous studies, we used local injection of a BMMSC suspension in a rat periodontitis model and found tissue defects were repaired [40]. Local injections of MSCs demonstrated its therapeutic potential in tissue regeneration by promoting host tissue remodeling [44–47]. The main advantage of MSC injection is that MSCs can be applied to the periodontal bone defects using minimally invasive surgeries.

“Cell sheet engineering” [48–50] was designed to avoid the shortcomings of traditional tissue engineering. When cultured MSCs are harvested as intact sheets along with their deposited extracellular matrix (ECM), they can be easily attached to host tissues with minimal cell loss. They also maintain cell-to-cell and cell-to-ECM connections, which are generally required to re-create functional tissues. The preservation and generation of ECM are helpful for tissue regeneration. Moreover, cell sheet implantation circumvents the use of scaffolds, preventing the strong inflammatory responses that biodegradable scaffolds would have incurred. In our previous study [12], we developed a simple and practical procedure to obtain PDLSC sheets via a Vc-mediated approach. In this study, new alveolar bone and periodontal soft tissues were regenerated to nearly normal levels 12 weeks after the implantation of hDPSC sheets. However, it requires open flap surgery which is traumatic for patients. Thus, such treatment is more suitable in combination with surgical periodontal treatment.

## Conclusions

This study supports the concept of using xenogeneic DPSCs cultured under GMP guidelines as a potential stem cell technology for periodontitis. Our data demonstrate that both xenogeneic DPSC sheets and DPSC injection can be appropriate therapies for periodontal bone and soft tissue regeneration.

## Abbreviations

AL, attachment loss; bFGF-2, basic fibroblast growth factor-2; CEJ, cemento-enamel junction; CFU-F, colony forming unit fibroblasts; CT, computed tomography; DPSC, dental pulp stem cell; ECM, extracellular matrix; EMD, enamel matrix derivative; GMP, good manufacturing practice; GTR, guide tissue regeneration; H&E, hematoxylin and eosin; HA-TCP, hydroxyapatite/tricalcium phosphate; hDPSC, human dental pulp stem cell; MSC, mesenchymal stem cell; PD, probing depth; PDLSC, periodontal ligament stem cell; SD, standard deviation; SEM, scanning electron microscopy; S-N-K, Student-Newman-Keuls test; STL, stereolithography; TEM, transmission electron microscopy; TGF-β, transforming growth factor-β; Vc, vitamin C

## Additional files

Additional file 1: Figure S1. Characterization of human dental pulp stem cells (hDPSCs) and the multi-differentiation potentials of hDPSCs. (A) Representative phase contrast microscopic photographs of hDPSCs after 14 days; the cultured hDPSCs from single colonies showed typical fibroblast-like cells under a light microscope. (B) Alkaline phosphatase activity, an early marker for osteo/dentinogenic differentiation, could be induced in hDPSCs (Test). (C) Oil red O-positive lipid clusters in hDPSCs indicated their adipogenic differentiation potential (Test). (D) Flow cytometry analysis of hDPSCs showed expression of cell markers CD73, CD105, CD90, and CD146, but not HLA-DR and CD45. (TIF 5686 kb) Additional file 2: Figure S2. Clinical operation of hDPSC injection. (A) Clinical assessments of the experimental periodontal bone defect immediately after osteotomy. (B) Intraoral photograph indicated the injection process of hDPSCs. (C) As demonstrated on a three-dimensional model, the suspension of hDPSCs was directly injected in the bottom of the alveolar bone defect area. (D) CT image showed the location of injected hDPSCs (*red arrow*). *B* bone, *hDPSC* human dental pulp stem cell. (TIF 4466 kb) Additional file 3: Figure S3. Three-dimensional CT imaging examination of bone regeneration. (A) CT Data were stored using the Dicom 3.0 standard and Dicom format default images were introduced into Mimics software 10.01. (B) Threshold values were set according to the Bone Scale in Mimics. Three-dimensional models of examined sites were reconstructed using Optimal, a setting in Mimics. (C) Three-dimensional model of one site. An ASCII stereolithography (STL) file of the bone was imported into Geomagic Studio, and excess parts beside the bone defect were roughly removed. (D) Cutted three-dimensional mode before and 12 weeks after operation of the same site were imported into Geomagic Studio. (E) N point fitting (*n* > 5) was used to overlap the three-dimensional model of the same site before and 12 weeks after operation. (F) Fully overlapped three-dimensional model of the same site before and 12 weeks after operation; extra areas beside bone defects were removed in accordance with the same parameters. Bone regeneration volume was then outputted. *Grey model*: Three-dimensional model before operation. *Blue model*: Three-dimensional model 12 weeks after operation. (TIF 3799 kb)
