# Supplementary figures and images for: Periodontal regeneration in swine after cell injection and cell sheet transplantation of human dental pulp stem cells following good manufacturing practice
Source: Stem Cell Res Ther. 2016 Sep 9;7(1):130. doi: 10.1186/s13287-016-0362-8 (PMC5017121; doi:10.1186/s13287-016-0362-8)

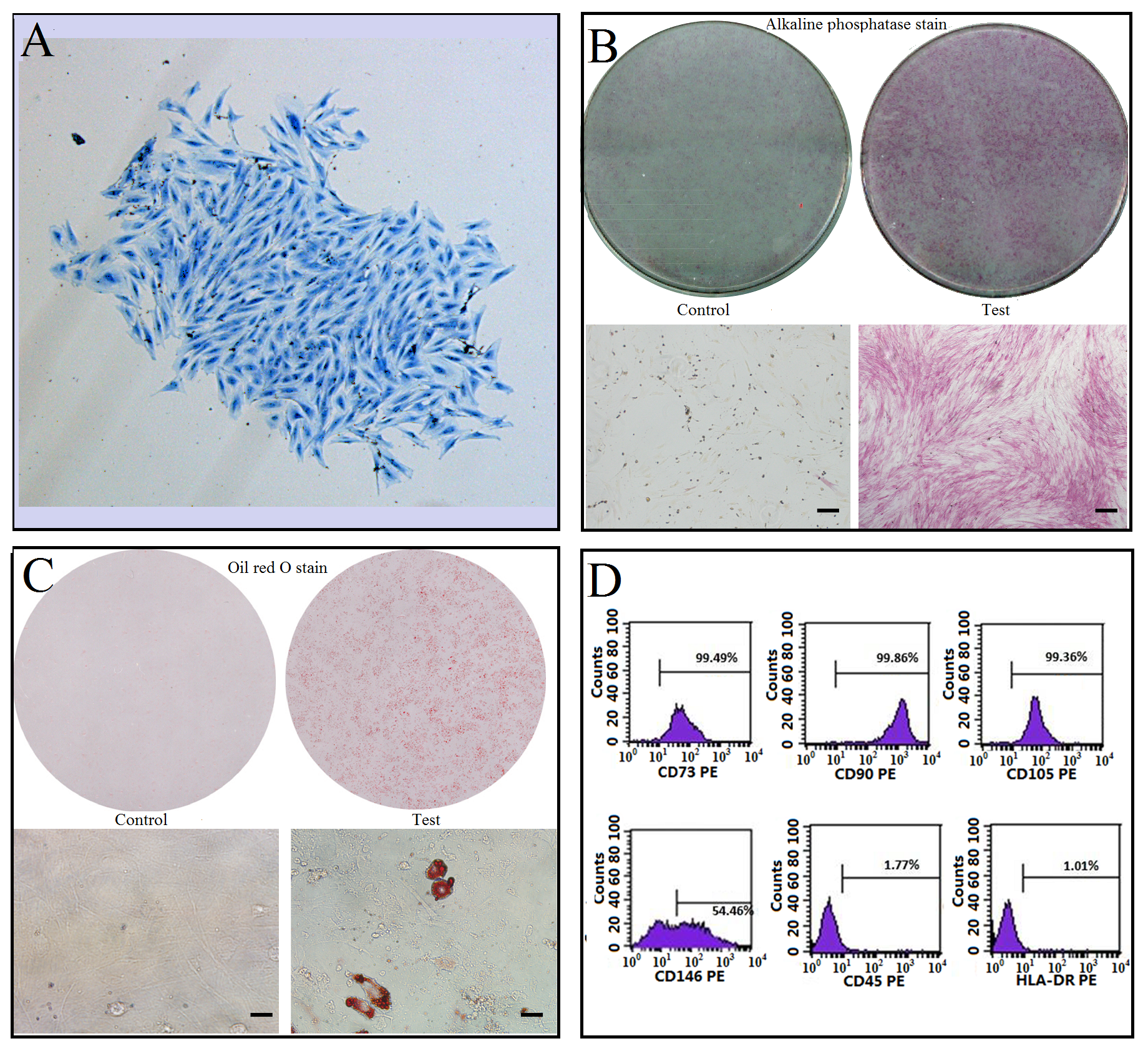

Supplement: Additional file 1: Figure S1. — Characterization of human dental pulp stem cells (hDPSCs) and the multi-differentiation potentials of hDPSCs. (A) Representative phase contrast microscopic photographs of hDPSCs after 14 days; the cultured hDPSCs from single colonies showed typical fibroblast-like cells under a light microscope. (B) Alkaline phosphatase activity, an early marker for osteo/dentinogenic differentiation, could be induced in hDPSCs (Test). (C) Oil red O-positive lipid clusters in hDPSCs indicated their adipogenic differentiation potential (Test). (D) Flow cytometry analysis of hDPSCs showed expression of cell markers CD73, CD105, CD90, and CD146, but not HLA-DR and CD45. (TIF 5686 kb) [file 13287_2016_362_MOESM1_ESM.tif]

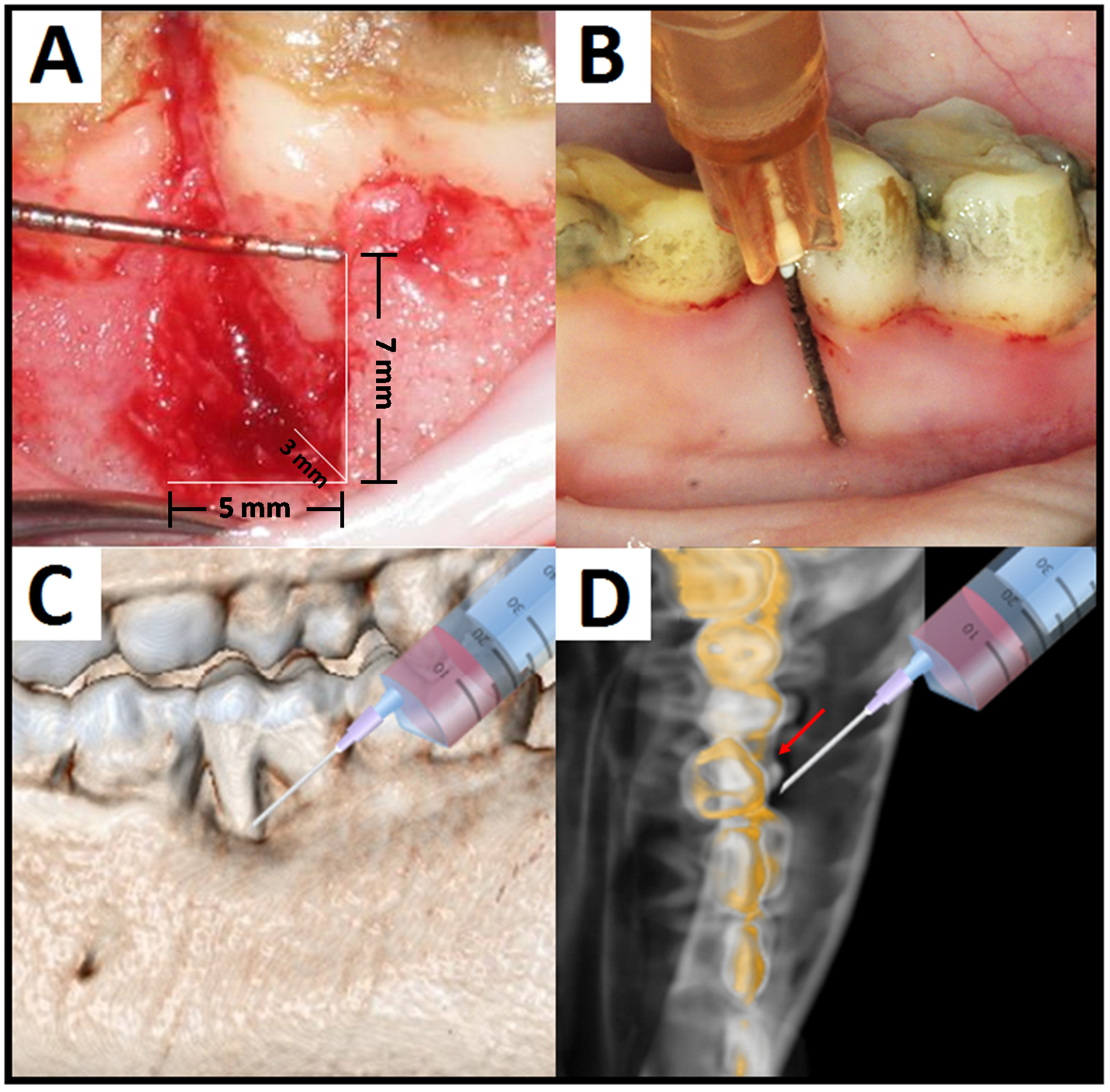

Supplement: Additional file 2: Figure S2. — Clinical operation of hDPSC injection. (A) Clinical assessments of the experimental periodontal bone defect immediately after osteotomy. (B) Intraoral photograph indicated the injection process of hDPSCs. (C) As demonstrated on a three-dimensional model, the suspension of hDPSCs was directly injected in the bottom of the alveolar bone defect area. (D) CT image showed the location of injected hDPSCs (red arrow). B bone, hDPSC human dental pulp stem cell. (TIF 4466 kb) [file 13287_2016_362_MOESM2_ESM.tif]

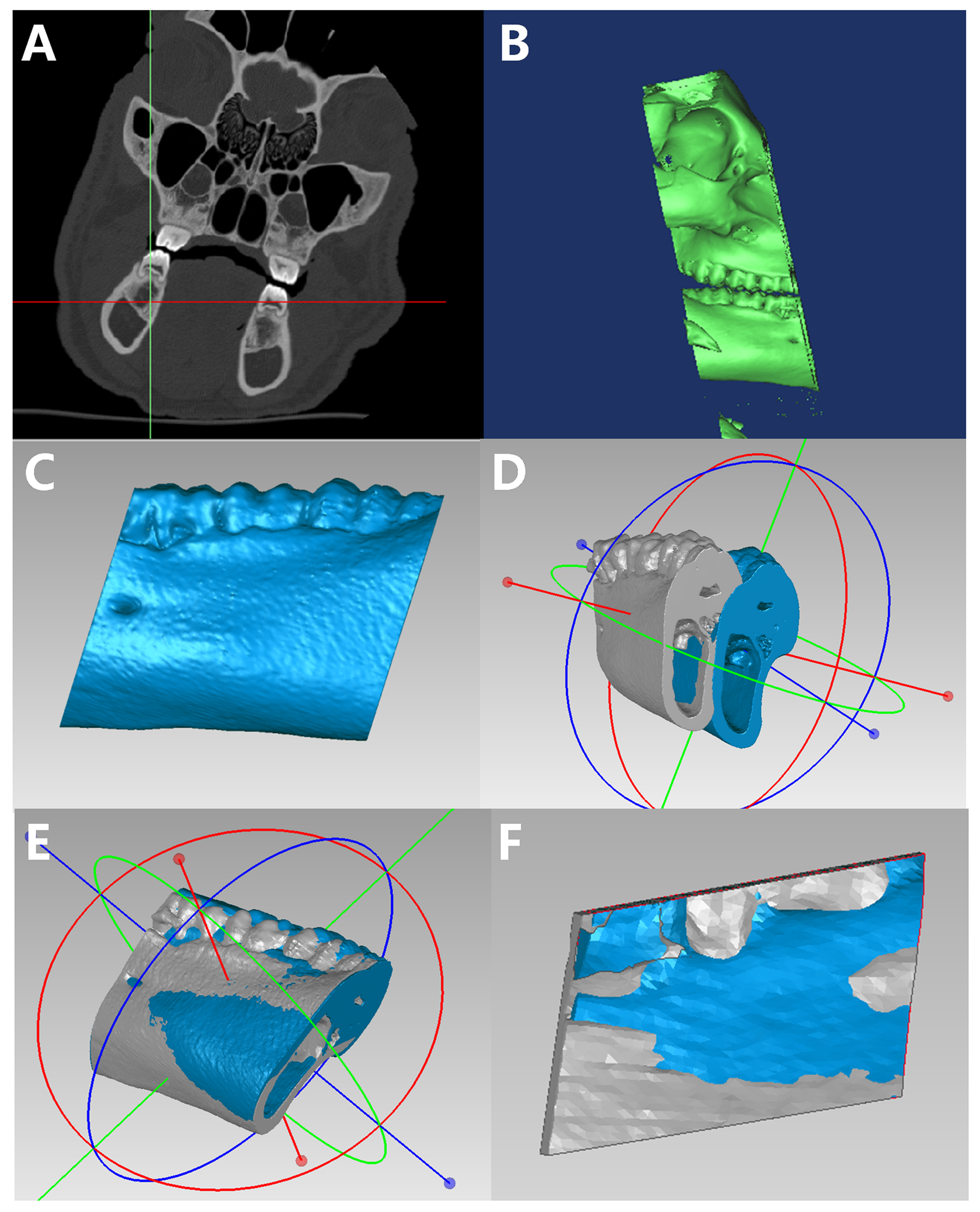

Supplement: Additional file 3: Figure S3. — Three-dimensional CT imaging examination of bone regeneration. (A) CT Data were stored using the Dicom 3.0 standard and Dicom format default images were introduced into Mimics software 10.01. (B) Threshold values were set according to the Bone Scale in Mimics. Three-dimensional models of examined sites were reconstructed using Optimal, a setting in Mimics. (C) Three-dimensional model of one site. An ASCII stereolithography (STL) file of the bone was imported into Geomagic Studio, and excess parts beside the bone defect were roughly removed. (D) Cutted three-dimensional mode before and 12 weeks after operation of the same site were imported into Geomagic Studio. (E) N point fitting (n > 5) was used to overlap the three-dimensional model of the same site before and 12 weeks after operation. (F) Fully overlapped three-dimensional model of the same site before and 12 weeks after operation; extra areas beside bone defects were removed in accordance with the same parameters. Bone regeneration volume was then outputted. Grey model: Three-dimensional model before operation. Blue model: Three-dimensional model 12 weeks after operation. (TIF 3799 kb) [file 13287_2016_362_MOESM3_ESM.tif]
